# An Analysis Through to Congruence Between Real and Self-Perceived Body Mass Index in Nursing Students

**DOI:** 10.3390/nursrep14040225

**Published:** 2024-10-21

**Authors:** Marta López-Bueno, Silvia Navarro-Prado, Ángel Fernández-Aparicio, Miriam Mohatar-Barba, María López-Olivares, Carmen Enrique-Mirón

**Affiliations:** 1 Department of Nursing, Faculty of Health Sciences, Melilla Campus, University of Granada, 52005 Melilla, Spain; martalopez@ugr.es (M.L.-B.); silnado@ugr.es (S.N.-P.); miriamb@ugr.es (M.M.-B.); 2 Instituto de Investigación Biosanitaria (ibs.GRANADA), 18014 Granada, Spain; 3 Department of Nutrition and Food Science, Faculty of Health Sciences, Melilla Campus, University of Granada, 52005 Melilla, Spain; mlopezolivares@ugr.es; 4 HUM-613 Research Group, Department of Inorganic Chemistry, Faculty of Health Sciences, Melilla Campus, University of Granada, C/Santander s/n, 52005 Melilla, Spain; cenrique@ugr.es

**Keywords:** nursing students, body mass index, obesity, perception of body weight, health promotion, prevention

## Abstract

Background/objectives: Overweight and obesity are growing concerns that also affect nursing staff, healthcare professionals that play a critical role in public health awareness and intervention. This study aimed to define the health parameters associated with body weight, analyze if there is an erroneous self-perception of overweight/obesity through the distortion of body weight perception, and determine the predictive factors of body weight distortion. Methods: A cross-sectional study of 224 nursing students gathered anthropometric and demographic data. Self-perceived body weight was assessed using Stunkard and Stellar’s scale. Descriptive statistics and multinomial logistic regression identified significant predictors of weight distortion. Results: The analysis found that men reported greater weight discrepancies than women. Specifically, 57% of the men (28 of 49 participants) underestimated their real BMI, while only 23% of the women (40 of 175 participants) did so. Age, sex, and weight classification emerged as significant explanatory variables for the distortion of body weight perception. Conclusions: The findings indicate a significant vulnerability among nursing students to the misperception of their own body weight status, highlighting the need for targeted training strategies. These strategies should focus on correcting misperceptions of obesity among health professionals throughout their working life to improve future obesity prevention efforts for society.

## 1. Introduction

From the 1980s to the present, the prevalence of obesity has experienced an alarming increase in high-income countries [1]. Despite efforts to contain this situation, the prevalence of obesity is also expected to increase in countries with fewer economic resources, due to the acquisition of new habits diverging from the traditional ones. In addition, by 2035, over 4 billion people worldwide are expected to be overweight or have obesity [2]. These data are especially concerning because obesity represents one of the main public health challenges in the world, not only because of its magnitude and severity, but also because of its role as a risk factor of different kinds of cancers, cardiovascular diseases (CVD) and type 2 diabetes mellitus [3].

Obesity is a global issue, with a prevalence of 65% in the general population. Unfortunately, overweight and obesity also affect healthcare professionals, including nurses, which make up a significant portion of the healthcare field. A research study performed in the United States of America (USA) found that more than half of the nursing staff were living with overweight or obesity (54.5%) [4]. Another study carried out in Poland found that the prevalence of obesity in nurses was 44% [5]. Additionally, an international study involving nurses from the United Kingdom, New Zealand and Australia revealed a higher prevalence of overweight and obesity in this professional group than in the general population [6]. Another study accomplished in Scotland by Kyle et al. [7], in which both healthcare and non-healthcare professionals participated, found a prevalence of overweight and obesity of 69.1% in nurses, a higher percentage than that observed in the rest of the professionals studied.

Traditionally, in several studies reporting the prevalence of overweight and obesity, body mass index (BMI), an indirect marker of adiposity, has been used in the categorization of body weight (BW) [8,9]. However, it is of importance to not only investigate the prevalence of obesity, but also the possible causes that lead to it. In this sense, some authors have pointed out that when overweight becomes a common phenotype, then an erroneous perception of obesity is produced [10]. For this reason, studying whether there is a false perception of obesity could provide valuable and necessary information to decelerate the progression of obesity. Likewise, studying this false perception among healthcare professionals is also necessary to ensure their maximum involvement in preventive activities.

In view of the above, the scale proposed by Stunkard and Stellar [11] allows us to determine the existing discrepancies between real and self-perceived BMI, and, therefore, to obtain the distortion of the BW variable in the studied population. Previous research has revealed that women are more likely to misperceive their BW than men. Specifically, women tend to overestimate their BW, and men tend to underestimate their BW [12,13,14,15,16]. At the same time, taking into account that obesity is a long-standing public health problem, it is necessary to study these issues in future healthcare professionals, since they will be the ones who will lead the future prevention of obesity.

For all of the above reasons, this study aims to determine if there is a misperception of overweight/obesity in a group of nursing students using the distortion of BW as a variable, and to determine the predictive factors of distortion of BW and its strength.

## 2. Materials and Methods

### 2.1. Study Design

This is a descriptive and cross-sectional observational study.

### 2.2. Participants and Recruitment

Participants in this study were recruited in 2021, through intentional sampling, among the Nursing Degree students of the Health Sciences Faculty of a city located on Africa’s north coast belonging to the University of Granada (Spain). During first-, second- and third-year practical classes, they were thoroughly informed about the study and encouraged to participate. To be included in the study, participating Nursing Degree students aged 18–35 years had to be healthy and free from any pre-diagnosed metabolic disorders or physical dysfunctions. Also, students had to sign the informed consent document. Students who did not meet these criteria were excluded from participation.

### 2.3. Instruments

**Figure 1:**
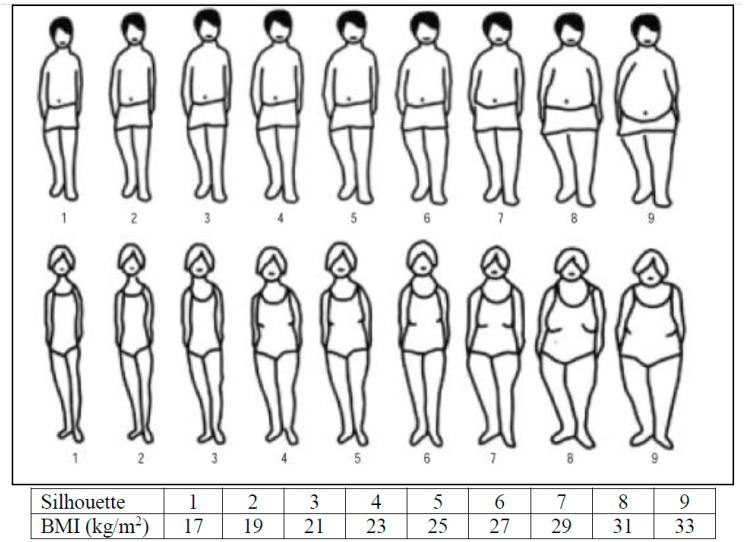
Stunkard and Stellar scale for assessing self-perceived body mass index.

According to the International Society for the Advancement of Kinanthropometry (ISAK) statements [17], an anthropometric assessment was performed on each participant by a level 2 anthropometrist certified by the ISAK. Anthropometric data were measured using a portable height rod and an impedance scale, model BC-601 (Tokyo, Japan), both from the TANITA brand. Demographic data were collected through a questionnaire. Self-perceived BMI was obtained by employing the scale proposed by Stunkard and Stellar [11] (Figure 1), later modified by Collins [18], which includes nine drawings of female and male figures. Each drawing represents different body mass index (BMI) values, from 17 kg/m^2^ to 33 kg/m^2^, in two-unit increments.

### 2.4. Process

After all participants completed the informed consent form, the demographic questionnaire and the Stunkard and Stellar scale were delivered by a member of the research team, and answers were recorded. Participants indicated on the Stunkard and Stellar scale the drawing they felt most identified with, in order to know the perceived BMI. Subsequently, height, weight and body composition were measured, and participants received feedback about their real body weight once the real BMI was known. BMI was calculated as BW divided by height squared (kg/m^2^) [19]. Moreover, participants were categorized according to the World Health Organization [19] and the Spanish Society for the Study of Obesity (SEEDO) criteria [20] as follows: underweight was defined by a BMI < 18.5 kg/m^2^, normal weight by a BMI from 18.5 to 24.99 kg/m^2^, overweight by a BMI from 25 to 29.99 kg/m^2^, and obesity by a BMI ≥ 30 kg/m^2^.

As reference values of the percentages of body fat (BF) in men and women aged 18–35 years old, we employed the values defined by the impedanciometer manufacturer. In the case of women, the healthy range is from 21% to 33%, and in men, it is between 9 and 20% [21].

Distortion of BW was determined through calculating the differences between real BMI and self-perceived BMI. Five categories were defined:

- Category 1: real BMI—self-perceived BMI: <−4 (overestimated their BMI);
- Category 2: real BMI—self-perceived BMI: between −4 and −2 (overestimated their BMI);
- Category 3: real BMI—self-perceived BMI: between −2 and 2 (perceived as they were; the BMI of the chosen shape was similar to the actual BMI obtained by anthropometry, with perception adjusted to their BMI);
- Category 4: real BMI—self-perceived BMI: between 2 and 4 (underestimated their BMI);
- Category 5: real BMI—self-perceived BMI: >4 (underestimated their BMI).

In this study, as in the one carried out by Soto et al. [22] at University of Navarra (Spain), categories 1 and 2 and categories 4 and 5 were also grouped, to facilitate the differentiation between those who overestimated, those who underestimated and those who had a more adjusted perception of their BMI.

### 2.5. Data Analysis

For statistical analysis, firstly place, data distribution was analyzed using the Kolmogorov–Smirnov test, and a normal distribution was not found in any of the variables (*p* < 0.001). Next, a description of the sample (means, standard deviation and frequencies) reported in Table 1 was provided through univariate analysis. Comparison of sociodemographic and anthropometric variables according to the misperception of body weight was performed by bivariate analysis (Chi-square and Mann–Whitney U, depending on the variables’ nature) with a statistical significance level of *p <* 0.05. Finally, multivariate analysis was performed through multinomial logistic regression to identify the predictive factors that may influence the discrepancies between real and self-perceived BMI (distortion of BW), and to obtain a strength of association estimate, adjusted for the effect of the remaining independent variables included in the model. The selection procedure followed was sequential forward. The SPSS v21.0 package for Windows was used.

### 2.6. Ethical Considerations

The performance of this study was authorized by the Research Commission of the Health Sciences Faculty (approval code REGAGE23e00048658918). This study was conducted in strict compliance with the guidelines and ethical principles for medical research on humans established by the World Medical Association in the Declaration of Helsinki. Detailed information about the study goals and characteristics was provided to all of the participants, and they were required to provide a signed informed consent to take part in the study. In addition, in accordance with the General Regulations on Data Protection and with Organic Law 3/2018, of 5 December, on the protection of personal data and digital rights, anonymity of the participants was assured through using codes.

## 3. Results

### 3.1. Characteristics of the Participants

Table 1 shows the results of the descriptive analysis of the demographic and anthropometric variables and distortion of BW of the 224 participants. Of the total number of students enrolled in the Nursing Degree (*n* = 387), 57.9% (224) participated in the study. Most of the participants were first-year students (68.6%), followed by second-year students (26.3%), and, to a lesser extent, third-year students (4.9%) due to the temporary co-incidence with their internship in health centers. Of the 224 participants, 78.1% were women and the remaining 21.9% were men. The age was a mean of 20.23 ± 2.8 years and a median of 19 years, and was in a wide range of between 18 and 35 years. The percentage distribution of professed religion was 72.8% (163) for Christians, 24.6% (55) for Muslims and 2.7% (6) for others. The percentage obtained in relation to sex and professed religion is representative of the enrolled students.

In relation to anthropometric variables, the average real BMI found in women (23.4 ± 4.9 kg/m^2^) was within the normal weight group, but not in the case of men, whose mean was within the overweight range, 25.5 ± 4.9 kg/m^2^. Those values, corresponding to the percentage of body fat, for men and women, are within the normal range. Also, for men and women, more than half of the sample (64.7%) correctly identified with the anatomical drawing representing their BMI. Of the remaining participants, 30.4% underestimated their BW and 4.9% overestimated it.

### 3.2. Body Weight Discrepancies in Nursing University Students According to the Sociodemographic Variables of Reference

Sex, age and real BMI variables are statistically significantly related to the distortion of BW variable (*p* < 0.05), this variable being independent of the degree year and religion professed (Table 2). Regarding sex, the percentage of cases with distortion of BW was higher in men than in women. Specifically, 57% of the men (28 of 49 participants) underestimated their real BMI, while only 23% of the women (40 of 175 participants) did so.

In relation to age, discrepancies among real and self-perceived BMI were higher as age increased. Finally, regarding the classification of BW according to real BMI, an underestimation of BMI was observed in more than half of the participants defined as overweight (57%), while this underestimation was observed in all of the participants classified as having obesity.

### 3.3. Body Weight Discrepancies According to the Anthropometric Variables of Reference in Nursing University Students

Non-compliance with the assumption of normality of the data (in all cases, *p* < 0.001) was verified through the Kolmogorov–Smirnov test, and Table 3 shows the mean values (±SD) of the anthropometric variables according to the different categories of the distortion of BW variable and the significance of the comparison of means, based on the Kruskal–Wallis test. For all studied variables, except for height and fat percentage, there were differences that were statistically significant in the distortion of BW. Thus, the means were lower or slightly the same when the BW was overestimated (except for age), and, conversely, they were higher when it was underestimated, except for the percentage of body water.

### 3.4. Multinomial Logistic Regression to Determine the Predictive Factors of Discrepancies Between Real BMI and Self-Perceived BMI

A multinomial logistic regression analysis was carried out to find out the possible distortion of BW predictive factors and their strength (Table 4). In this model, distortion of BW was considered a dependent variable (response) and entered into the model as a factor, taking “underestimated” as a reference category. According to the results obtained in the descriptive and two-dimensional analyses, the variables considered explanatory were sex (categorized 1-Male and 2-Female, the latter being the reference category), weight status according to BMI (categorized 1-Insufficient weight, 2-Normal weight, 3-Overweight and 4-Obesity, the reference category being 4-Obesity) and age, taken in this case as a continuous quantitative variable.

In the multinomial logistic regression model obtained (Table 4), the interaction of the variables sex, age and weight status (according to BMI) resulted as an explanatory variable, providing, in this sample and for a specific age, the probability of classifying participants according to sex and weight status, in each of the defined categories for the self-perceived BMI. Thus, the probability of having an overestimated BW compared to an underestimated one almost tripled (2.285) in women who were underweight, for each unitary increase in age (β = 1.046; 95% CI, 2.277–3.555; *p* < 0.001). For overweight women, the probability of having an underestimated BW increased by five-year increments of age, compared to women with an adjusted weight (β = −0.230; 95% CI, 0.686–0.920; *p* = 0.002), increased sixfold (6.300). In men with normal weight, it was by every five-year increment in their age that the probability of having an underestimated perception of their BW increased sixfold (6.100), compared to those with an adjusted probability (β = −0.201; 95% CI, 0.709–0.944; *p* = 0.006), while in the same time frame and in those who were overweight, the probability of having this perception (β = −0.289; 95% CI, 0.637–0.880; *p* < 0.001) increased almost sevenfold (6.650).

## 4. Discussion

Nursing students will constitute a key element in the prevention of obesity in the future. Therefore, it is important to prevent and identify overweight and obesity in order to reduce health risks in this group [23]. The objectives of our study aim to define the health parameters associated with BW in a group of nursing students, as well as to analyze them through the misperception of body mass index. In our study we analyzed, through the distortion of BW, the presence of a misperception of overweight/obesity in a group of nursing students, and we also determined the predictive factors of distortion of BW and its strength. The main findings of our study are as follows: (i) men reported greater weight discrepancies than women; (ii) age, sex, and weight classification emerged as significant explanatory variables for the distortion of body weight perception.

In the present study, more than half of the participants were normo-weight (64.7%), 23.7% had overweight, and 6.3% had obesity. These data are similar to those found in a study developed in Germany, in which researchers compared two groups of nursing students, in two different phases. In the second phase, which took place five years later than the first one, they found that 24.3% of participants had overweight, and 7.3% had obesity, numbers that turned out to be higher than those in the data found in the first phase [24]. However, other studies report more worrying data that reveal that almost half of future nurses already have this health problem. An example is shown by a study carried out in Europe, specifically in Scotland, in which an overweight prevalence of 29% and obesity prevalence of 18.2% were found [25]. This reality was also observed in other research performed in non-European countries, such as one carried out in South Africa, where the authors found that 49.7% of the studied population had overweight and obesity, and one in Brazil, with a similar finding, 52.6% [26,27]. All the above-mentioned data support the evidence of the increasing obesity prevalence in nursing students in the next few years.

The reported findings in the present study with regard to the distortion of BW variable unveiled a misperception of real BMI in 35.4% of participants, observing showing 30.4% of participants underestimated their real BMI, while 4.9% overestimated it. Also surprising was the underestimation of real BMI found in slightly more than half of the students classified as overweight (57%); even more surprising was the underestimation of all the participants classified with obesity. Similarly, other studies have also found misperceptions of BMI in participants with overweight and obesity. Thus, in a study carried out in Poland, 21.2% of participants with overweight or obesity did not recognize these pathologies, with men being the ones who showed a greater lack of knowledge of their real BMI. This same study revealed a higher cardiovascular risk in participants who underestimated their BMI than among those who perceived themselves correctly [28]. Other research developed in Australia evaluated the ability of patients and family physicians to recognize overweight and obesity. In total, 26.8% of patients with overweight or obesity did not self-perceive in accordance with their actual BMI. Regarding the recognition ability of the physicians, 60.8% of the overweight patients and 60% of the patients with obesity were correctly identified by them. Based on these results, the authors concluded that inaccurate perception impeded the recognition of overweight and obesity by patients and also by family physicians. In addition, they noted that the increasing prevalence of overweight and obesity could be a contributing factor to the lack of recognition of overweight and obesity [29]. Therefore, some of the findings found in this study can be explained by factors such as the social environment, since several authors suggest that living with people with obesity could influence the lack of awareness of obesity as a real health problem [30,31,32].

As for the relationship found between the age variable and the self-perceived BMI, the results agree with those of other studies performed on nursing students. In a study that took place in Kuwait in which 202 nursing students participated, it was observed that the prevalence of overweight and obesity increased as the age of the participants increased. This study reported a prevalence of overweight and obesity of 26.2% and 11.9%, respectively [33]. Another investigation carried out in the USA, in which 233 undergraduate and 230 postgraduate students participated, found statistically significant differences in the self-reported BW according to the academic level (*p* < 0.001). In this study, the mean BMI of the undergraduate students (27.8 ± 7.2 kg/m^2^) was slightly higher than that of the postgraduate students (25.5 ± 5.7 kg/m^2^). In addition, the percentage of postgraduate students who self-reported to have obesity (33%) was significantly higher than that of younger ones (22%). It should be noted that the mean age of the undergraduate students was 20.4 ± 5.2 kg/m^2^, and that of the postgraduate students was 36.1 ± 8.4 kg/m^2^ [34].

Regarding the association of the sex variable with the misperception of the real BMI, significant differences were found between men and women (*p* < 0.001), highlighting that more than half of the men (57%) underestimated their real BMI. In addition, the multinomial logistic regression analysis showed that men, as they become older, are more likely to underestimate their real BMI than women, although women also underestimate their real BMI. It should be added that other studies in which the Stunkard and Stellar silhouettes were used also found a greater underestimation of the BW in older participants [35,36,37].

Health promotion and education is one of the main strategies to address a complex and multifactorial problem such as obesity. Therefore, it is essential to prevent healthcare professionals from having an inaccurate perception of obesity that prevents them from recognizing it, as shown in a recent article. In this article, the underestimation of obesity by healthcare personnel prevented the establishment of adequate treatment because excess BW was not considered in the diagnosis [38]. Taking into account that nurses, in the context of prevention, are more likely to discuss health behavior when they perceive that it is important and deserves appropriate attention [38], it would be a priority to avoid the misperception of obesity in these professionals. Hence, it is advocated that nurses should project a healthy image, i.e., they should act as health role models for the general population. However, there are contrary opinions that assert that the acquisition of competencies, skills, and confidence are the factors that ensure the efficiency of health promotion, regardless of the nurse’s own health behavior [39,40].

Our study presents strengths and limitations. Among its strengths, it should be noted that non-self-reported anthropometric measurements were used, giving greater accuracy. As limitations, we highlight the sample size, and that the majority of the sample were young women. Another limitation was that BMI was used in our study, but this index presents a poor discriminatory variable among individuals with similar BMIs, but with different risk profiles [8,9]. For this reason, results should be interpreted with caution.

## 5. Conclusions

In conclusion, in a context where the prevalence of obesity is increasing at a worrisome rate, the present investigation is useful for demonstrating the special vulnerability of nursing students to overweight and obesity, as well as the presence of an erroneous perception of obesity, which could be related to the increase in its prevalence. In addition, the study revealed the possible existence of a potential problem that may increase as the age of health professionals increases. Therefore, it is necessary to implement strategies aimed at training nursing professionals on determining the correct association between actual BMI and self-perceived BMI throughout their professional life. Also, it is essential to provide continuing education for health professionals to ensure the acquisition of competencies, and for them to develop the ability to recognize obesity as a pathological condition, in order to implement effective preventive measures to halt the advance of this long-standing health problem.

## Figures and Tables

**Table 1:** Sociodemographic and anthropometric data of nursing university students (*n* = 224).

Variables	Values
*Demographic Data*	
Sex (%)	
Male	21.9
Female	78.1
Age (%)	
18–19 years old	52.2
20–22 years old	32.6
>22 years old	15.2
Course (%)	
First year	68.3
Second year	26.3
Third year	4.9
Professed religion (%)	
Christian	72.8
Muslim	24.6
Other	2.7
*Anthropometric data (mean ± SD)*	
Weight (kg)	65.5 ± 14.0
Male	80.4 ± 17.4
Female	61.4 ± 9.5
Height (cm)	165.1 ± 8.2
Male	177 ± 6.1
Female	162 ± 5.2
BMI	23.8 ± 3.8
Male	25.5 ± 4.9
Female	23.4 ± 3.4
Body fat percentage	27.0 ± 7.6
Male	19.4 ± 7
Female	29.2 ± 6.3
Muscle mass (kg)	45.1 ± 9.5
Male	60.4 ± 8.1
Female	40.8 ± 3.8
Body water percentage	53.4 ± 6.3
Male	56.3 ± 9.9
Female	52.7 ± 4.6
*Weight classification (according to real BMI) (%)*	
Under weight (<18.5)	3.6
Normal weight (18.5–24.9)	66.5
Overweight (25.0–29.9)	23.7
Obesity (≥30)	6.3
*Distortion of body weight (%)*	
Overestimated	4.9
Adjusted	64.7
Underestimated	30.4

SD: standard deviation, BMI: body mass index, kg: kilograms, cm: centimeter.

**Table 2:** Distortion of body weight according to the sociodemographic variables of reference in nursing university students (*n* = 224).

Variables	*n*	Distortion of Body Weight (%)			Chi-Square	*p*
		Overestimated	Adjusted	Underestimated		
*Sex*						
Male	49	0	43	57	22.540	<0.001
Female	175	6	71	23		
*Age (years old)*						
18–19	117	4	73	23	10.256	0.03
20–22	73	4	62	34		
>22	34	9	44	47		
*Course*						
First year	153	7	65	28	3.945	0.41
Second year	59	2	66	32		
Third year	11	0	55	45		
*Professed religion*						
Christian	163	6	64	31	1.329	0.86
Muslim	55	4	65	31		
Others	6	0	83	17		
*Weight classification (according to actual BMI)*						
Underweight	8	25	75	0	73.924	<0.001
Normal weight	149	6	78	16		
Overweight	53	0	43	57		
Obesity	14	0	0	100		

BW: body weight; BMI: body mass index. Level of significance *p*: 0.05. Chi-square test of independence.

**Table 3:** Anthropometric variables of reference in nursing university students according to the distortion of body weight (*n* = 224).

Anthropometric Variables	Distortion of Body Weight, Mean ± SD			Chi-Square	*p*
	Overestimated	Adjusted	Underestimated		
Age (years old)	21.1 ± 3.7	19.8 ± 2.2	20.9 ±3.6	5.469	0.04
Weight (kg)	56.3 ± 7.6	60.8 ± 8.8	77.1 ± 16.7	61.366	<0.001
Height (cm)	163.4 ± 5.5	164.1 ± 7.1	167.6 ± 10.2	3.473	0.18
Fat percentage	26.0 ± 6.6	26.4 ± 7.3	28.6 ± 8.2	3.698	0.16
Muscle mass (kg)	39.6 ± 2.7	42.3 ± 6.5	51.89 ± 11.9	43.442	<0.001
Bone mass (kg)	2.1 ± 0.2	2.5 ± 2.5	2.74 ± 0.6	41.717	<0.001
Water percentage	54.6 ± 5.4	54.4 ± 5.1	51.24 ± 8.0	9.540	0.008
Actual BMI	20.6 ± 2.6	22.5 ± 2.6	27.19 ± 4.1	68.740	<0.001

SD: standard deviation; BMI: body mass index; kg: kilograms; cm: centimeters. Level of significance *p*: 0.05, Chi-square test of independence.

**Table 4:** OR and IC 95% for the logistic regression analysis using underestimated perception of the body mass index as a reference (*n* = 224).

Distortion of BW	Gender	Weight Status According BMI	*p*	β	OR	95% CI
Overestimated	Women	Underweight	<0.001	1.046	2.845	2.277–3.555
Adjusted	Men	Normal weight	0.006	−0.201	0.818	0.709–0.944
		Overweight	<0.001	−0.289	0.749	0.637–0.880
	Women	Overweight	0.002	−0.230	0.795	0.686–0.920

OR, odds ratio; CI, confidence interval; BW, body weight; BMI, body mass index.

## Data Availability

The data presented in this study are only available on request from the corresponding author so that the privacy of participants can be maintained.

## Public Involvement Statement

Nursing students participated in the study; the type of participation, the type of data collected, and the collection procedure are reported in the methodology.

## Guidelines and Standards Statement

This manuscript was drafted in accordance with the STROBE Statement for cross-sectional research.
